# *SACCHAROMYCES CEREVISIAE* FUNGEMIA IN A PEDIATRIC PATIENT AFTER TREATMENT WITH PROBIOTICS

**DOI:** 10.1590/1984-0462/;2017;35;3;00014

**Published:** 2017-07-31

**Authors:** Mariá Ribas Romanio, Ligia Augusto Coraine, Vinicius Pignoti Maielo, Marcelo Luiz Abramczyc, Renato Lopes de Souza, Nilton Ferraro Oliveira

**Affiliations:** a Centro Universitário Cesumar, Maringá, PR, Brasil.; b Universidade Federal de São Paulo, São Paulo, SP, Brasil.

**Keywords:** Saccharomyces cerevisiae, Fungemia, Intensive Care Units, pediatrics, Child

## Abstract

**Objective:**

To report the case of a one-year-old patient with a bloodstream infection associated with probiotics, and to discuss the indications and precautions concerning the therapeutic use of probiotics.

**Case description:**

A one-year-old male patient with Down syndrome in a late postoperative period of congenital cardiac disease correction. The patient was severely malnourished and had been hospitalized since he was two months old in the Pediatric Intensive Care Unit. While in the hospital, the patient presented multiple infections related to mechanical ventilation and invasive devices, and received recurrent treatment with broadspectrum antibiotics for long periods. The patient developed chronic diarrhea and feeding intolerance, which lead to the use of probiotics (*Saccharomyces boulardii*) for four days. Two days after the end of the treatment, the patient developed septic shock, and the *Saccharomyces cerevisiae* was isolated in the central and peripheral blood cultures. After antifungal treatment (Amphotericin B), the blood cultures were negative. The patient had no further clinical complications after this event.

**Comments:**

Despite the well-documented benefits of probiotics in some clinical situations, we should be cautious about the indication of their use, preparation, and administration, in addition to the safe handling of invasive devices.

## RESUMO

**Objetivo:**

Descrever um paciente com infecção de corrente sanguínea associada ao uso de probiótico em criança de um ano de idade e discutir as principais indicações e precauções com o emprego terapêutico desses microrganismos.

**Descrição do caso:**

Paciente masculino, um ano de idade, portador de síndrome de Down, em pós-operatório tardio de correção de cardiopatia congênita, com desnutrição grave e internado desde os dois meses de vida em Unidade de Terapia Intensiva Pediátrica. Durante o período de internação, apresentou inúmeras infecções relacionadas à ventilação mecânica, cateteres vasculares e outros dispositivos, com uso prolongado e recorrente de antibióticos de amplo espectro. Evoluiu com diarreia crônica e intolerância alimentar, que culminou com o uso de probiótico (*Saccharomyces boulardii*) por quatro dias. Dois dias após o término do probiótico, desenvolveu choque séptico, com hemoculturas central e periférica positivas para *Saccharomyces cerevisiae*. Após tratamento antifúngico (Anfotericina B), houve negativação das culturas. O paciente evoluiu sem complicações clínicas adicionais após o evento.

**Comentários:**

Apesar dos benefícios bem documentados do uso de probióticos em algumas situações clínicas, deve-se ter cautela quanto à indicação de uso, preparo e administração do medicamento, além do manuseio seguro dos dispositivos invasivos do paciente.

## INTRODUCTION

Probiotics are live microorganisms, which may confer benefits to patients if administered in adequate doses. The benefits include the prevention and treatment of diarrhea through the use of antibiotics and the improvement of inflammatory bowel disease through immunomodulation, among others.^1^ ^,^ ^2^

One of the most commonly used probiotics is *Saccharomyce*s *boulardii*, a *Saccharomyces cerevisiae* strain that innocuously colonizes the respiratory, genitourinary, and intestinal human systems. *Saccharomyces cerevisiae* can be pathogenic and cause systemic infection from the microorganism itself in debilitated or immunosuppressed patients, especially when used as probiotic for treatment of diarrhea caused by antibiotics.^3^ Cases of the use of probiotics immediately preceding or concomitant to the occurrence of fungaemia by *Saccharomyces cerevisiae* are reported in literature. One of the main factors associated with this fungaemia is the presence of a central venous catheter.^2^ ^,^ ^4^ ^,^ ^5^ Its use in humans has several well-documented clinical indications, such as inflammatory bowel disease, malabsorption syndrome, prevention and treatment of diarrhea secondary to enteral or parenteral nutrition, and prophylaxis for infection from *Clostridium difficile.* ^6^ ^,^ ^7^

The purpose of this article is to describe a case of bloodstream infection associated with the use of probiotics in children, and to discuss their indications and precautions.

## CASE REPORT

A one-year-old male patient with Down syndrome was in a late postoperative period of congenital cardiac disease correction. The patient had hypothyroidism and severe chronic malnutrition and had been hospitalized in a pediatric intensive care unit (PICU) since he was two months old.

After surgical cardiac disease correction, the patient evolved with limited nutritional reestablishment and recovery because of intolerance to enteral feeding and multiple infections associated with mechanical ventilation and invasive devices. He received broadspectrum antibiotics in a recurrent and prolonged manner. Therefore, the use of probiotics (Saccharomyces*boulardii -* 200 mg capsules) was indicated for four days at a dose of 200 mg, 12/12 h, via a nasogastric tube.

Two days after having finished receiving probiotics, the patient developed septic shock, resulting in the collection of cultures and the expansion of the antibiotic spectrum for vancomycin, ciprofloxacin, and amphotericin B. Initially, the central venous catheter was not replaced because of the small numbers of sites available (multiple strokes and previous phlebotomy). After five days of expanding antimicrobial therapy, and with the child stable from a clinical standpoint, the blood cultures (central and peripheral) identified yeast in a partial result, without the growth of aerobic bacteria. At that moment, Vancomycin and Ciprofloxacin were suspended and Amphotericin B was replaced by Fluconazole, with the hypothesis of *Candida* infection sensitive to Fluconazole.

After changing the therapeutic regimen, the patient’s clinical condition deteriorated, leading to an empirical extension of Amphotericin B, Vancomycin and carbapenem, in the absence of new blood culture results. Due to this extension, there was clinical and laboratory improvement of the infectious condition. The final identification of blood cultures, which were taken from central and peripheral sites, occurred on the seventh day of treatment and showed the presence of *Saccharomyces cerevisiae,* without available antifungigram. This result led to the discontinuation of antibiotics on the tenth day of prescription and the maintenance of Amphotericin B for up to 14 days after obtaining negative blood cultures taken at the beginning of the extended antimicrobial therapy. The patient had a central venous catheter and nasogastric tube, which were replaced after the identification of *Saccharomyces cerevisiae*. The peritoneal dialysis catheter and the tracheostomy tube were not replaced. It is worth noting that, at that moment, no other patient in the inpatient unit presented a *Saccharomyces cerevisiae* infection*.*

The patient remained hemodynamically stable, in nutritional rehabilitation therapy. The patient’s guardian signed the informed consent form and the institution’s Research Ethics Committee approved the report.

## DISCUSSION

There are reports in the literature of systemic *Saccharomyces cerevisiae* infection, especially in adults; however, there are few cases among children.^5^ ^,^ ^8^ The infection can occur via two routes: intestinal translocation and venous catheter contamination, both from health care workers handling the medication with their hands, or the aerial drift of the strains after the capsules are opened. There are reports of infected individuals not only among those who received treatment with probiotics, but also in patients who shared the room with another patient undergoing treatment. Some studies showed that viable strains could be detected up to one meter away from the site of manipulation and persisted on the surface after two hours. Thus, it is therefore more prudent to handle them away from patients.^4^ Moreover, the *Saccharomyces cerevisiae* strains remain on the hands of professionals who handled them without gloves, even after appropriate cleaning.^9^

The incidence of fungemia from *Saccharomyces cerevisiae* is unknown and in most reports occurs isolated, although some cases of endocarditis, liver abscesses, and disseminated disease have already been described. There are few descriptions of fungaemia from *Saccharomyces cerevisiae* in previously healthy patients, and the main risk factor is the patient’s use of probiotics or their use by other individuals admitted to the same unit in nearby locations. Furthermore, infection associated with the presence of a central venous catheter is reported.^4^

The patient had severe malnutrition, with limited weight gain and bloating, which prevented progress in his diet. The patient also repeatedly used broad-spectrum antibiotics for bloodstream infections, which increased the chance of *Clostridium difficile* infection*.* For these reasons, the use of probiotics was prescribed. Moreover, this patient was using several invasive devices such as a tracheostomy, a peritoneal dialysis catheter, and a central venous catheter and he was considered immunosuppressed because of severe malnutrition. Therefore, the patient had numerous risk factors for disseminated infection because of the probiotic.

The unit where the patient was hospitalized is a level III ICU, which provides general pediatric care at a university hospital. It has a lounge area with nine beds separated by curtains, an isolation room, and no single rooms, in compliance with the requirements of Resolutions 7 and 50 of *Agência Nacional de Vigilância Sanitária* (ANVISA).^10^ ^,^ ^11^ Medication is prepared in the same environment where patients are hospitalized, but more than one meter away from all beds. The team wears gloves to prepare and administer the medication and they wash their hands. Because no other patient had an infection caused by the same agent at that time, and the necessary precautions for the preparation and administration of the medication were followed, the authors believe there was intestinal translocation of the yeast in this case. The dose of the probiotic was indicated on the medication package leaflet approved by ANVISA.

The treatment indicated for bloodstream infection associated with the use of probiotics is the removal of the contaminated central venous catheter and the use of Amphotericin B 1 mg/kg/day or Fluconazole 10 mg/kg/day, although some articles describe strains resistant to Fluconazole and even to Amphotericin B.^4^ An article concerning the fungi colonization in hematologic patients revealed *Saccharomyces cerevisiae* with reduced sensitivity to fluconazole, although none of these patients have developed an invasive disease. In Spain, three cases of concomitant fungaemia caused by *Saccharomyces cerevisiae* were reported in an Intensive Care Unit (ICU) and isolated strains also showed reduced sensitivity to Fluconazole (MIC 8 mcg / dL). Some authors attribute this sensitivity spectrum to the frequent use of Fluconazole in the ICU and in patients with multiple comorbidities, and to the transmission of this agent through the hands of professionals. There are also reports of patients who developed fungemia caused by *Saccharomyces cerevisiae* during the use of Fluconazole (or prophylactic treatment of candidemia). Nevertheless, the outcome is usually favorable and similar to the outcome found from the use of the two antifungals.^8^ ^,^ ^12^ ^,^ ^13^

It is important to note that this report presents limitations due to the unavailability of the antifungigram of the isolated agent that is used to guide therapy, and molecular analysis to confirm that it is the same yeast as the probiotic administered. However, other clinical studies in similar cases showed that the *Saccharomyces cerevisiae* found in the patient culture and the *Saccharomyces boulardii* of the probiotic administered were genetically identical.^4^ ^,^ ^9^

It can be concluded that despite the well-documented benefits of probiotics, their use should be carefully evaluated for each patient because of the risk of bloodstream infection, especially in immunosuppressed individuals. The preparation of probiotics outside the patient’s room, and the use of gloves is recommended.^6^ One should pay attention to the correct dosage, as well as to the manipulation and preparation of the probiotics in order to avoid the contamination of other patients and the patient’s own invasive devices, such as central venous catheters and probes.

## References

[1] Vandenplas Y, Huys G, Daube G (2015). Probiotics: An update. J Pediatr.

[2] Herbrecht R, Nivoix Y (2005). Saccharomyces cerevisiae fungemia: an adverse effect of Saccharomyces boulardii probiotic administration. Clin Infect Dis.

[3] Llanos R, Querol A, Pemán J, Gobernado M, Fernández-Espinar MT (2006). Food and probiotic strains from the Saccharomyces cerevisiae species as a possible origin of human systemic infections. Int J Food Microbiol.

[4] Muñoz P, Bouza E, Cuenca-Estrella M, Eiros JM, Pérez MJ, Sánchez-Somolinos M (2005). Saccharomyces cerevisae fungemia: an emerging infectious disease. Clin Infec Dis.

[5] Belet N, Dalgiç N, Oncel S, Ciftçi E, Güriz H, Barlas M (2005). Catheter-related fungemia caused by Saccharomyces cerevisiae in a newborn. Pediatr Infect Dis J.

[6] Hennequin C, Kauffmann-Lacroix C, Jobert A, Viard JP, Ricour C, Jacquemin JL (2000). Possible role of catheters in Saccharomyces boulardii fungemia. Eur J Clin Microbiol Dis.

[7] Vandenplas Y, Salvatore S, Vieira M, Devreker T, Hauser B (2007). Probiotics in infectious diarrhoea in children: are they indicated?Eur. J Pediatr.

[8] Thygesen JB, Glerup H, Tarp B (2012). Saccharomyces boulardii fungemia caused by treatment with a probioticum. BMJ Case Rep. Epub.

[9] Lherm T, Monet C, Nougière B, Soulier M, Larbi D, Le Gall C (2002). Seven cases of fungemia with Saccharomyces boulardii in critically ill patients. Intensive Care Med.

[10] Brasil. Ministério da Saúde. Agência Nacional de Vigilância Sanitária (2010). Resolução RDC nº 7 de 24 de fevereiro de 2010. Dispõe sobre os requisitos mínimos para funcionamento de Unidades de Terapia Intensiva e dá outras providências. Diário Oficial da União.

[11] Brasil. Ministério da Saúde. Agência Nacional de Vigilância Sanitária (2002). Resolução RDC nº 50 de 21 de fevereiro de 2002. Dispõe sobre o Regulamento Técnico para planejamento, programação, elaboração e avaliação de projetos físicos de estabelecimentos assistenciais de saúde. Diário Oficial da União.

[12] Salonen JH, Richardson MD, Gallacher K, Issakainen J, Lehtonen OP, Nikoskelainen J (2000). Fungal colonization of haematological patients receiving cytotoxic chemotherapy: emergence of azole-resistant Saccharomyces cerevisiae. J Hosp Infect.

[13] Enache-Angoulvant A, Hennequin C (2005). Invasive Saccharomyces Infection: a comprehensive review. Clin Infect Dis.

